# A novel ferroptosis-related gene signature for predicting outcomes in cervical cancer

**DOI:** 10.1080/21655979.2021.1925003

**Published:** 2021-05-14

**Authors:** Xingling Qi, Yipeng Fu, Jia Sheng, Meng Zhang, Mingxing Zhang, Yumeng Wang, Guiling Li

**Affiliations:** a Department of Integration of Western and Traditional Medicine, Obstetrics and Gynecology Hospital of Fudan University, Shanghai, China; b Department of Breast Surgery, Obstetrics and Gynecology Hospital of Fudan University, Shanghai, China; c Department of Nursing, Obstetrics and Gynecology Hospital of Fudan University, Shanghai, China

**Keywords:** Cervical cancer, prognosis, biomarker, the cancer genome Atlas, differentially expressed genes

## Abstract

Ferroptosis, a newly discovered iron-dependent form of cell death, contributes to various pathologies; however, the prognostic value of ferroptosis-related genes (FRGs) in cervical cancer (CC) remains unclear. Herein, we identified 15 differentially expressed FRGs based on data from The Cancer Genome Atlas database. Ten FRGs that correlated with prognosis were screened by univariate Cox regression analysis. The least absolute shrinkage and selection operator regression model was performed to develop a novel prognostic signature. A four-gene model was built to separate samples into high-risk and low-risk groups. Overall survival was lower in the high-risk group than in the low-risk group (*p* < 0.05). Receiver operating characteristic curve showed a good diagnostic efficiency of the signature. The risk score was identified as an independent prognostic factor via multivariate Cox regression. A functional analysis further revealed a difference in the immune status between the two risk groups. To conclude, we constructed a novel prognostic signature based on FRGs. Targeting ferroptosis may represent a promising approach for the treatment of CC.

## Grapical Abstract

**Figure:**
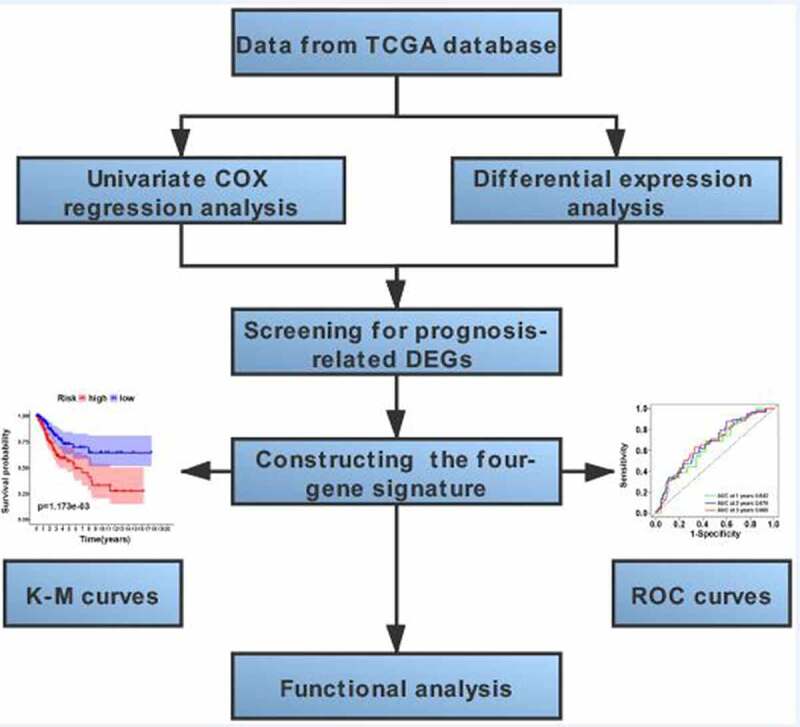

## Highlights

- Fifteen differentially expressed genes in cervical cancer were identified.
- A novel ferroptosis-related four-gene signature was built.
- Ferroptosis may be associated with prognosis and immunity in cervical cancer.

## 1. Introduction

Cervical cancer (CC) ranks fourth in both prevalence and mortality among malignancies in women worldwide [1], accounting for approximately 570,000 new cases and 311,400 deaths per year [2]. Human papilloma virus (HPV) infection is the main cause of CC [3]; however, CC only develops in a minority of HPV carriers. CC is a complex and heterogeneous disease involving genetic and environmental factors, making the prediction of outcomes a challenge. Patients with CC have a poor prognosis with limited treatment options, especially those at an advanced stage or with recurrent disease [4]. Despite the availability of cervical screening programs and HPV vaccines, CC remains an important public health issue worldwide [5]. The accurate identification of patients at a high risk of poor prognosis and timely adjustment of treatment strategies, including the use of immunotherapy or targeted therapy, may improve survival. Therefore, it is necessary to find reliable biomarkers and to develop novel prognostic models for CC.

Cell death is the terminal fate of individual cells and is an indispensable part of homeostasis [6]. Tumor initiation and development are related to a reduction in cell death. Therefore, studies of tumor cell death are expected to provide a rationale for the design of therapeutic targets. Apoptosis, autophagy, aponecrosis, pyroptosis, and necrosis are major cell death mechanisms [7]. Ferroptosis is a recently identified mode of programmed cell death in which the iron-dependent accumulation of reactive oxygen species and lipid peroxidation trigger death [8,9]. It constitutes a constellation of unique genetic, biochemical, and morphological features, different from other types of regulated cell death [10]. Recent studies have revealed that ferroptosis is involved in many pathophysiological processes, such as kidney injury [11], neurodegenerative diseases [12], T-cell immunity [9], and metabolic diseases [13]. In addition, ferroptosis has been observed in various malignancies, such as ovarian cancer [14], liver cancer [15], glioma [16], osteosarcoma [17], and renal cell carcinoma [18]. Interestingly, dependence on iron and sensitivity to ferroptosis are greater in cancer cells than in healthy cells. Therefore, ferroptosis may be an alternative target for therapy-resistant cancer treatment [19]. In addition to drug-induced cell ferroptosis, a number of genes associated with tumor formation and progression have also been identified as markers or modulators of ferroptosis. For example, *RRM2* protects against ferroptosis by sustaining GSH synthesis in liver cancer cells [15], and *SCD1* facilitates tumor growth, protects against ferroptosis, and is associated with a poor prognosis in gastric cancer [20]. These ferroptosis-related biomarkers are promising drug targets for the precision treatment of various cancers. However, associations between ferroptosis-related genes (FRGs) and prognosis in CC have not been evaluated.

We hypothesized that FRGs are associated with prognosis and tumor immunity in CC. To evaluate this hypothesis, we conducted a series of analyses of FRGs in CC, including univariate Cox regression, LASSO regression, multivariate Cox regression, and functional enrichment analyses. We established an innovative prognostic gene signature for predicting clinical outcomes. Our results provide new insight into the role of ferroptosis in CC and provide a basis for the development of novel targeted anticancer drugs.

## 2. Materials and methods

### 2.1. Raw data acquisition and pre-processing

RNA sequencing (RNA-seq) expression data (level 3) and corresponding clinicopathological information for 309 samples were acquired from The Cancer Genome Atlas (TCGA) portal up to 8 April 2020 (https://portal.gdc.cancer.gov/projects). All gene expression data were normalized using the across-array scale function of the limma R/Bioconductor package (http://www.r-project.org). Gene expression values were converted to fragments per kilobase of transcript per million mapped reads (FPKM). All datasets analyzed in this study are publicly available (TCGA data portal); therefore, approval from an external ethics committee was not required. Additionally, our research was in strict adherence to TCGA data access policies and publication guidelines. A list of 60 ferroptosis-associated genes was derived from previous literature [19,21–23] and is presented in Supplementary STable 1.

### 2.2. Construction of a FRG-based prognostic model

First, the limma Bioconductor R package was utilized to identify differentially expressed genes (DEGs) between CC samples and adjacent non-cancerous cervical tissue samples. The false discovery rate (FDR) threshold was set at *p* < 0.05 for DEG calling. Second, the univariate Cox regression method was used to screen for FRGs with potential prognostic significance. To control for false discovery, all *p* values were adjusted with the Benjamini–Hochberg (BH) correction algorithm. Then, a protein–protein interaction (PPI) network of proteins encoded by all overlapping DEGs with prognostic value was visualized using String (http://string-db.org) (version 11.0) [24]. To avoid overfitting, the LASSO L1-penalized Cox regression method was utilized to select variables with high prognostic value [25,26]. Next, 1000 LASSO iterations were performed for prognostic model construction using the ‘glmnet’ package in R.

The final prognostic model included the normalized candidate gene expression matrix as the independent variable. The overall survival (OS) time and patient survival status were considered response variables. The optimal regularization parameter λ was selected by 10-fold cross-validation at one standard error, where the final λ parameter value reached the classification error of cross-validation. The risk score for all samples was thus estimated by the normalized gene expression levels and corresponding regression coefficients in the model. The established formula for calculating risk scores was as follows: Risk score = ∑n_i_ = ∑Coef_i_ × x_i_, where x_i_ represents the normalized expression level of target gene i and Coef_i_ represents the regression coefficient. All patients were further allocated into high- or low-risk cohorts based on the median value of the risk scores. PCA was performed using the ‘prcomp’ function in the STATS package in R based on expression levels of genes in the signature. A t-SNE analysis was implemented to compare distributions between the two cohorts using the R package Rtsne (https://github.com/jkrijthe/Rtsne). For a survival analysis of the genes in the signature, the best cutoff point was iteratively determined with the function surv_cutpoint in the survminer package (version 0.4.6). To evaluate the performance of the prognostic signature in differentiating patients with CC with distinct risk levels, time-dependent ROC curves were generated using the R package survivalROC (version: 1.0.3).

### 2.3. Functional enrichment and pathway analysis

Gene Ontology (GO) and Kyoto Encyclopedia of Genes and Genomes (KEGG) pathway enrichment analyses for all selected DEGs between the two risk cohorts were performed with the clusterProfiler package in BioConductor using |log2FC| ≥1 and FDR <0.05 as thresholds. The *p* values were then adjusted by the BH correction method. Enrichment scores for 16 tumor-infiltrating immune cells as well as the activity of 13 signaling pathways related to immune response were obtained by a single-sample gene set enrichment analysis (ssGSEA) implemented in the Bioconductor R package ‘GSVA’ [27].

### 2.4. Statistical analysis

The Student’s *t*-test was used to compare gene expression levels between CC samples and non-cancer cervical samples. Differences in proportions were evaluated using the chi-squared (χ^2^) test. Then, to compare ssGSEA enrichment scores for immune cells and immune-related pathways between the two groups (i.e., high- or low-risk groups), the Mann–Whitney U test was used. The OS for the two risk groups was evaluated by the log-rank test (Mantel-Cox) and Kaplan–Meier (KM) survival curves. To determine independent prognostic factors, both univariate and multivariate survival analyses with the Cox regression model were implemented. All statistical analyses were completed using R (Version 3.6.3) and SPSS (Chicago, IL, USA; Version 20.0). Unless otherwise stated, *p* < 0.05 was considered statistically significant. The *p* values for all analyses were determined from two-tailed tests.

## 3. Results

Targeting ferroptosis may provide a new therapeutic approach for tumors [28,29]. However, the role of FRGs in CC remains largely unknown. We evaluated the FRG signature and its association with survival in CC. In particular, we identified 10 prognostic FRGs by univariate Cox regression analysis. These hub genes were screened to establish a four-gene signature for predicting prognosis. KM survival analysis and ROC curves indicated the good predictive value of the signature. Furthermore, this signature was identified as an independent prognostic factor for CC survival in both univariate and multivariate analyses. We conducted a series of functional analyses of the FRGs, including a GO enrichment analysis, KEGG pathway analysis, and ssGSEA, revealing several relevant immune molecules and pathways.

**Figure 1.**
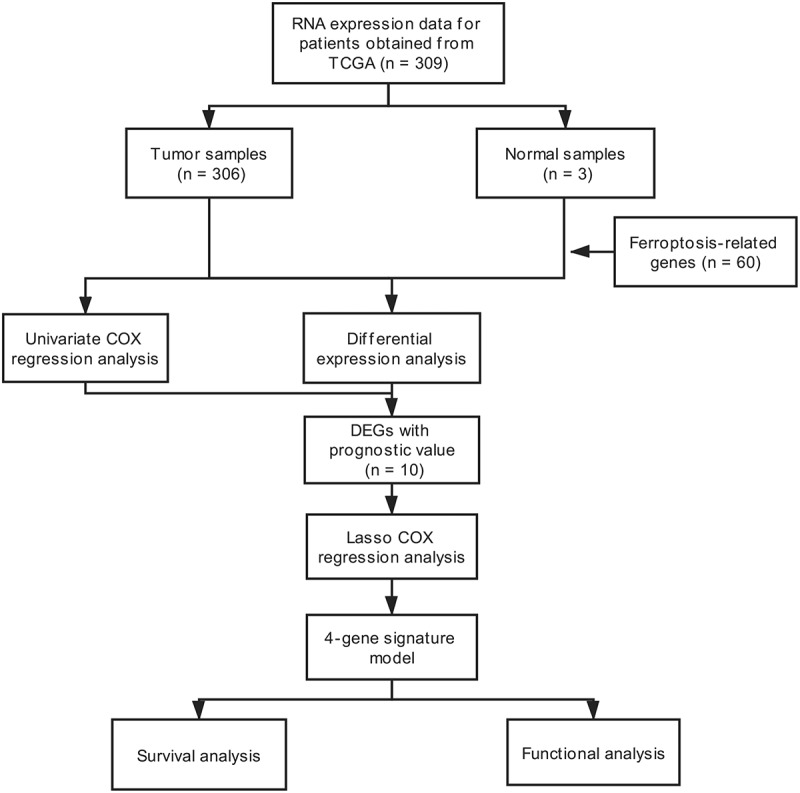
Flow-chart of patient enrollment and data collection

The workflow scheme for this study is illustrated in Figure 1. In total, 309 samples were included in analyses. The clinical and pathological characteristics of patients are reported in Table 1.

**Table 1.** Baseline characteristics of patients with cervical cancer

Parameter	Subtype	Number of cases (%)
**Age (years)**	≤50	179 (61.7)
	>50	111 (38.3)
**Histological grade**	I+ II	147 (50.7)
	III+IV	116 (40.0)
	Unknown	27 (9.3)
**Clinical stage**	I+ II	223 (76.9)
	III+IV	61 (21.0)
	Unknown	6 (2.1)
**Survival status**	Alive	220 (75.8)
	Dead	70 (24.2)

### 3.1. Selection of ferroptosis-related genes associated with survival

**Figure 2.**
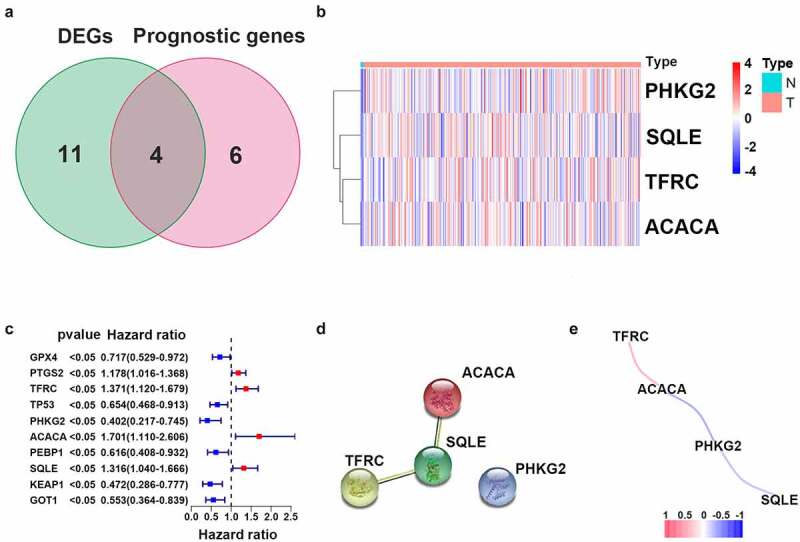
Identification of candidate genes related to ferroptosis in cervical cancer. (a) Venn diagram illustrating prognostic DEGs between cervical cancer and adjacent non-cancer samples. (b) Heatmap analysis of 10 prognostic DEGs. (c) Forest plot with hazard ratios from the survival analysis based on the univariate Cox regression model using gene expression levels as variables. (d) Construction and visualization of a protein–protein interaction (PPI) network of DEGs generated using the STRING database. Yellow lines represent text-mining evidence in the PPI network, and black lines represent co-expressed proteins. (e) Network analysis of internal correlations among four candidate genes. Correlation coefficients are indicated by different colors

To determine the specific expression patterns of FRGs, complete RNA-Seq datasets and corresponding CC clinical profiles in TCGA were examined. Among 60 FRGs, 15 genes were significantly differentially expressed between CC samples and adjacent cervical tissue samples. Ten genes (i.e., *GPX4, PTGS2, TFRC, TP53, PHKG2, ACACA, PEBP1, SQLE, KEAP1*, and *GOT1*) were statistically significantly related to OS in the univariate Cox regression model (Figure 2a). These were selected as hub genes for further analyses (FDR < 0.05; Figure 2(b, c)). A PPI network further demonstrated that *SQLE* was a hub gene (Figure 2d). Correlations between expression levels of genes are shown in Figure 2e.

### 3.2. Ferroptosis-related prognostic signature construction

**Figure 3.**
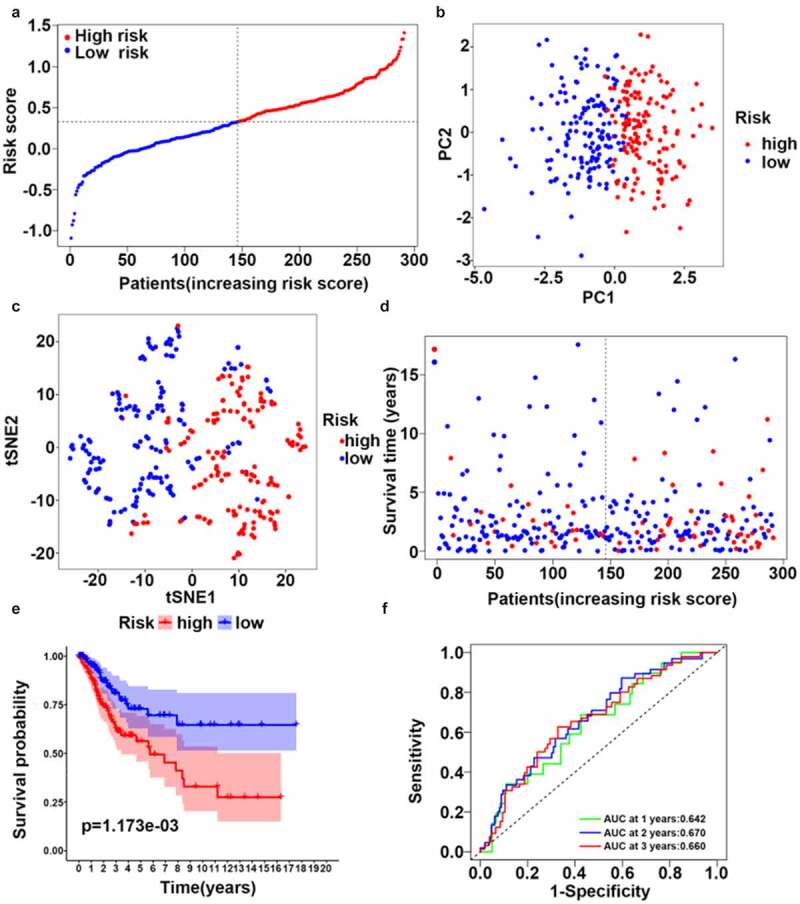
Synthetic analysis of the prognostic value of the ferroptosis-based risk signature. (a) Distribution of risk scores among CC samples. (b) Score plot for the principal component analysis (PCA). (c) Two-dimensional projection of CC-seq data from TCGA by a t-SNE analysis. (d) Distribution of four-gene risk scores and patient survival status. (e) OS by Kaplan–Meier curves for patients in the two risk groups. (f) Time-dependent ROC curves for survival prediction with AUC values

A multivariate LASSO cox regression model was used to build a prognostic signature based on the expression information for 10 ferroptosis-related hub genes. A four-gene ferroptosis-related signature was constructed according to the best setting for the tuning parameter λ. In survival analyses, the OS for patients with high gene expression levels was poor according to the best cutoff value for gene expression (adjusted *p* value < 0.05). The formula for the prognostic risk assessment score was as follows: risk score = (0.195 × *TFRC* expression values + 0.104 × *ACACA* expression values + 0.097 × *SQLE* expression values – 0.512 × *PHKG2* expression values). After samples with a survival time of zero were removed, all samples were allocated to the high-risk (n = 145) or low-risk score (n = 146) groups based on the median risk score (Figure 3a). Next, we deleted samples with no complete clinicopathologic information and explored risk stratification for patients with different clinicopathologic factors, including age, histological grade, and clinical stage. Age, histological grade, and clinical stage were all significantly correlated with a higher risk of poor outcomes (Table 2). PCA and t-SNE mappings showed that patients formed two distinct clusters (Figure 3(b, c)). As the risk score increased, the patients’ survival time decreased, and the death risk increased (Figure 3d). This was consistent with the results of the KM survival analysis (Figure 3e, *p* < 0.05). The prognostic efficiency of the four-gene signature was assessed by time-dependent ROC curves. The area under the curve (AUC) values were 0.642 (1-year), 0.670 (3-year), and 0.660 (5-year) (figure 3f).

**Table 2.** Baseline clinical characteristics of patients in the two risk groups

Characteristics	High risk	Low risk	p-value
**Age (%)**			**<0.001**
≤50 y	150 (48.8)	305 (99.3)	
>50 y	157 (51.2)	2 (0.7)	
**Histological grade (%)**			**<0.001**
I+ II	259 (84.3)	223 (72.6)	
III+IV	48 (15.7)	84 (27.4)	
**Clinical stage (%)**			**<0.001**
I+ II	166 (54.1)	302 (98.3)	
III+IV	141 (45.9)	5 (1.7)

### 3.3. Independent prognostic value of the four-gene signature

**Figure 4.**
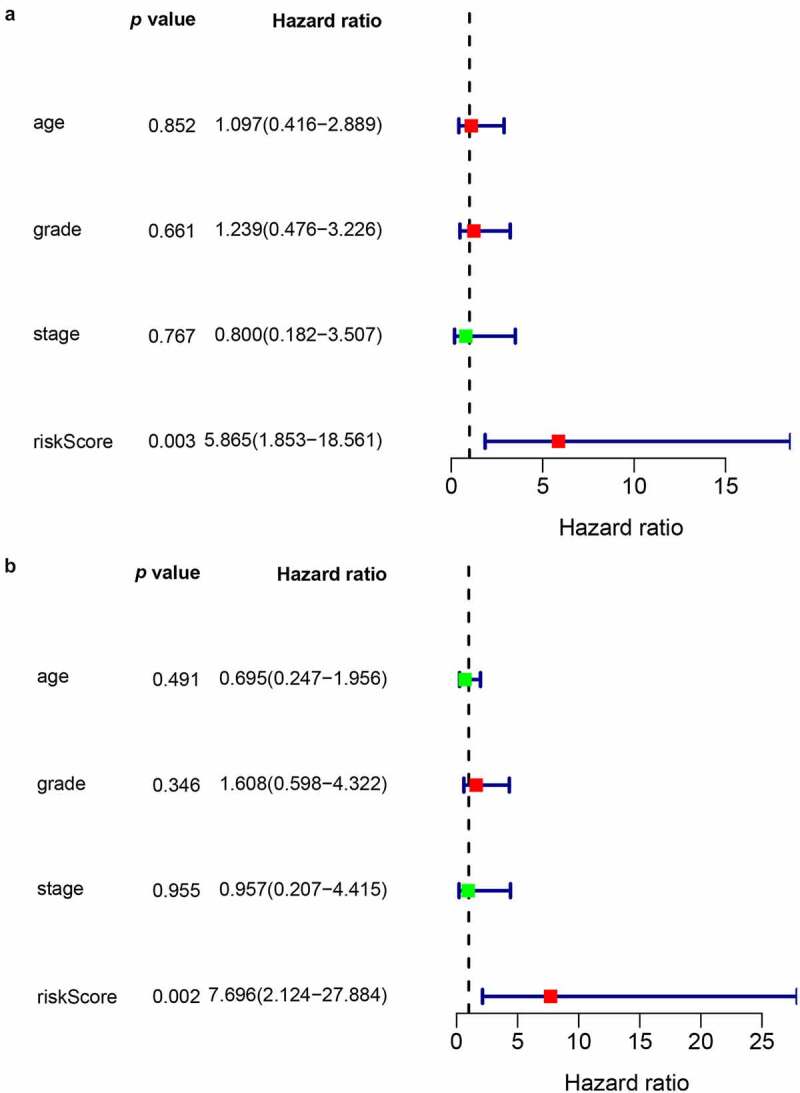
Prognostic value of the risk scores in the univariate (a) and multivariate (b) COX regression models

To examine whether the risk score could be an independent prognostic marker for CC, both univariate and multivariate Cox regression models based on the risk score and other clinical risk factors were used. A univariate analysis showed that the risk score was obviously related to OS (HR = 5.865, 95% CI = 1.853–18.561, *p* < 0.05) (Figure 4a). After adjustment for potential confounders, the risk score was still an independent indicator of survival in the multivariate analysis (HR = 7.696, 95% CI = 2.124–27.884, *p* < 0.05) (Figure 4b).

### 3.4. Functional analysis of the four-gene signature

**Figure 5.**
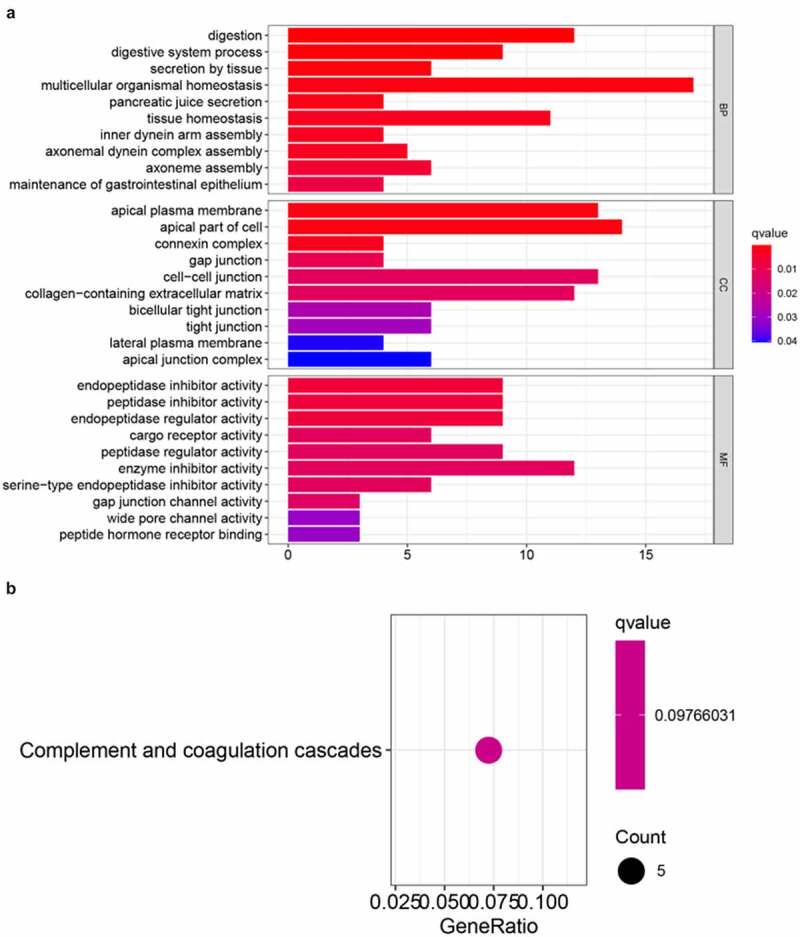
Results of GO (a) and KEGG pathways enrichment analyses (b) of DEGs among CC samples

To investigate potential biological pathways and functions related to the risk signature, GO annotation and KEGG pathway analyses of the DEGs between these two risk groups were conducted. According to the GO enrichment analysis, these DEGs were predominantly enriched in several biological processes, such as multicellular organismal homeostasis, digestion, tissue homeostasis, and iron secretion by tissue (adjusted *p* < 0.05, Figure 5a). Interestingly, the KEGG enrichment analysis indicated that these DEGs were significantly enriched in complement and coagulation cascades that are closely associated with inflammatory and immune responses (adjusted *p* < 0.05, Figure 5b).

**Figure 6.**
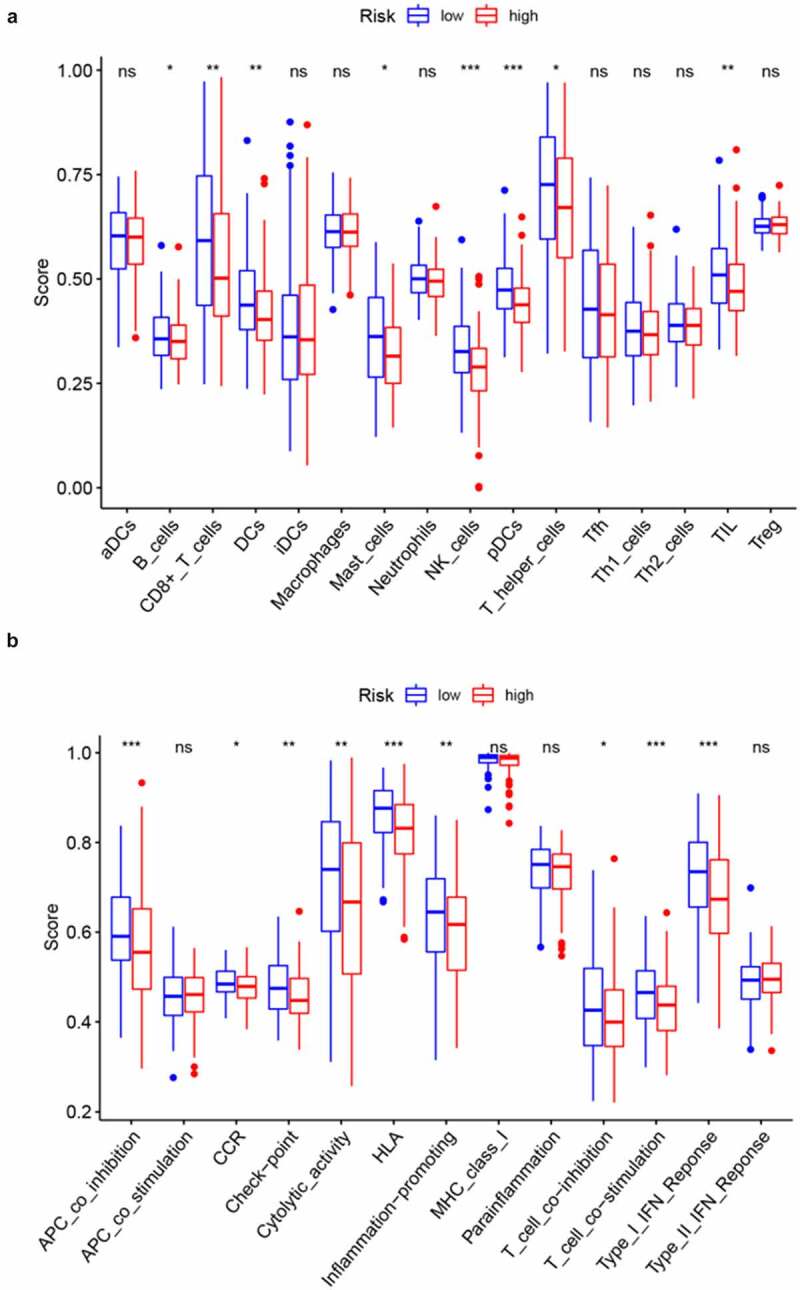
Comparison of ssGSEA enrichment scores between high- and low-risk groups in CC samples from TCGA. Boxplots display the distribution of ssGSEA enrichment scores of 16 immune cells (a) and 13 immune-related biological processes (b). CCR, cytokine-cytokine receptor. Adjusted p-values are represented as follows: ns, not significant; *p < 0.05; **p < 0.01; ***p < 0.001

To further probe the relationship between the prognostic signature and immune status, ssGSEA was conducted to obtain enrichment scores for several immune cell types as well as related biological pathways or functions. Interestingly, the contents of antigen processing and presentation, including the enrichment scores for DCs, pDCs, and APC co-inhibition differed significantly between the two risk groups (adjusted *p* value < 0.05, Figure 6a, b). Additionally, the scores for B cells, CD8 + T cells, Mast cells, T helper cells, NK cells, TIL, CCR, check-point, cytolytic activity, HLA, inflammation-promoting, T cell co-inhibition, T cell co-stimulation, and Type I IFN response were higher in the low-risk group than in the high-risk group (adjusted *p* value < 0.05, Figure 6(a, b)). Remarkably, the scores for pDCs and NK cells showed the most highly significant differences between the two risk groups.

## 4. Discussion

In this study, we comprehensively analyzed 60 FRGs in CC samples and their relationships with prognosis. We established a new ferroptosis-related four-gene signature. In functional analyses, these genes were significantly enriched in several immune cell types and immune-related pathways.

Although the potential regulatory effects of the inhibition of ferroptosis on the proliferation of CC cells has been reported [30], the mechanisms by which FRGs impact CC and OS remain poorly studied. We identified 15 FRGs that were significantly differentially expressed between CC samples and adjacent non-cancer samples. Unexpectedly, 10 of these genes (66.7%) were related to OS in a univariate Cox regression analysis, supporting the general importance of ferroptosis in the pathogenesis and progression of CC. These results also suggests that a predictive signature could be constructed using ferroptosis-related prognostic markers.

The proposed ferroptosis-related predictive signature generated in our study included four genes (*TFRC, ACACA, SQLE*, and *PHKG2*). *TFRC* encodes a transmembrane glycoprotein expressed on the surface of all nuclear cells; it facilitates iron uptake by combining with Fe^3+^-loaded transferrin for subsequent endocytosis. *TFRC*, an indispensable iron transporter for cellular iron absorption, is critical in carcinogenesis and tumor progression and is dysregulated in numerous cancers [31,32,32]. Additionally, it is closely related to invasion, clinical stage, and pelvic lymph node metastases in CC [33,34]. *ACACA*, which encodes a cytosolic enzyme involved in de novo fatty acid synthesis, exhibits biotin carboxylase and carboxyl transferase activity [35]. Its downregulation exerts an anti-tumor effect via the lipid metabolism pathway in colorectal cancer cells [36]. In addition, a novel compound CIL56 that can induce cell death via *ACACA* has been identified [37]. *SQLE* is a newly found ferroptosis regulator involved in cholesterol metabolism. *SQLE* promotes tumor progression by multiple mechanisms in different types of cancer cells [38]. It exerts an oncogenic effect on ALK+ anaplastic large cell lymphoma by the alteration of the membrane lipid composition and inhibition of ferroptosis [39]. However, the inhibition of *SQLE* expression rescues tumor growth defects in small cell lung cancer [40]. Therefore, the specific molecular mechanisms underlying the effects of the *SQLE* gene on ferroptosis in different tumors need to be further explored. *PHKG2*, which encodes the catalytic subunit of phosphorylase b kinase, could mediate the hormonal and neural regulation of glycogen breakdown (glycogenolysis) by a cascade of events, thereby activating glycogen phosphorylase. Previous studies have found that *PHKG2* expression is elevated in human breast cancer cells, indicating that it might be a useful diagnostic biomarker [41]. Additionally, it may be a potential biomarker for patients carrying wild-type KRAS in metastatic colorectal cancer [42].

In summary, three genes (*ACACA, SQLE*, and *PHKG2*) in the newly developed signature have established roles in the protection of tumor cells against ferroptosis, whereas *TFRC* has the opposite effect. Our results indicated that *ACACA* and *SQLE* are associated with short survival times in patients with CC, and *PHKG2* is positively correlated with prognosis. Therefore, the effects of these genes on survival via the regulation of ferroptosis-related signaling pathways and biological processes require further investigation. Other than *TFRC*, few empirical studies have evaluated the mechanism and biological effects of these FRGs.

Although the ferroptosis-related molecular mechanisms at the cellular level have recently become the subject of intensive tumor research, the precise relationship between ferroptosis and immunity remains unclear. Thus, we further conducted GO and KEGG enrichment analyses of the DEGs between low-risk and high-risk groups. As expected, these genes were mainly enriched in secretion by tissues, digestion, and tissue homeostasis, involving iron absorption and secretion. Surprisingly, our results indicated that these genes were primarily enriched in complement and coagulation cascades. Therefore, it is plausible that ferroptosis is highly correlated with tumor immunity.

Of note, a highly significant difference in the importance of genes related to antigen processing and presentation was observed between the two risk groups. One possible explanation for this difference is as follows. Ferroptotic dead or dying cells initiate lipid oxidation and generate pro-ferroptotic signals, including chemokines and lipid mediators, to drive active APCs to reach the site of ferroptosis [43]. In addition, when compared with the high-risk groups, patients in the low-risk groups had higher fractions of CD8 + T cells and NK cells. This directly demonstrates that CD8 + T cells and NK cells are correlated with a favorable prognosis in CC due to their ability to target and kill tumor cells [44], consistent with numerous previous studies [45–47]. Furthermore, low-risk scores were associated with the activation of the anti-tumor immune response, including the activity of tumor-infiltrating lymphocytes, HLA, T cell co-stimulation, and type I IFN response. Therefore, improved anti-tumor immunity activity in patients with CC at low risk might explain the favorable prognosis (Figure S1).

Despite these important findings, this study had various limitations. First, a retrospective study design based on TCGA was used. Thus, selection biases might limit the validity of this research, and further prospective studies are warranted to verify our conclusions using large-scale real-world data. Second, the prognostic model lacked external validation, which may pose a limitation in terms of generalizability. Finally, our model was constructed based on single FRGs. The integration of multiple markers of multiple clinical features into the signature would further improve the predictive value of the model.

## 5. Conclusion

This is the first systematic investigation of the expression patterns of FRGs in CC and their relationship with patient outcomes. A four-gene prognostic signature based on FRGs was developed. High-risk scores were associated with poor survival. Our results provide new insights into ferroptosis in cervical carcinogenesis and progression and offers a valuable ferroptosis-targeted therapeutic avenue for CC. Moreover, our results further support the role of FRGs in tumor immunity. Given that our work is based on TCGA RNA-Seq data, the specific mechanisms by which FRGs contribute to anti-tumor immunity should be further explored in the future.

## Supplementary Material

Supplemental Material

Supplemental Material

## Data Availability

All gene expression profiles and corresponding clinical-pathological data of cervical cancer patients can be downloaded from the TCGA online database.
