# Supplementary material for: A novel ferroptosis-related gene signature for predicting outcomes in cervical cancer
Source: Bioengineered. 2021 May 14;12(1):1813–25. doi: 10.1080/21655979.2021.1925003 (PMC8806749; doi:10.1080/21655979.2021.1925003)
Supplement: Supplemental Material [file KBIE_A_1925003_SM8659.docx]

**A novel ferroptosis-related gene signature for predicting outcomes in cervical cancer**

**Figure S1**

**
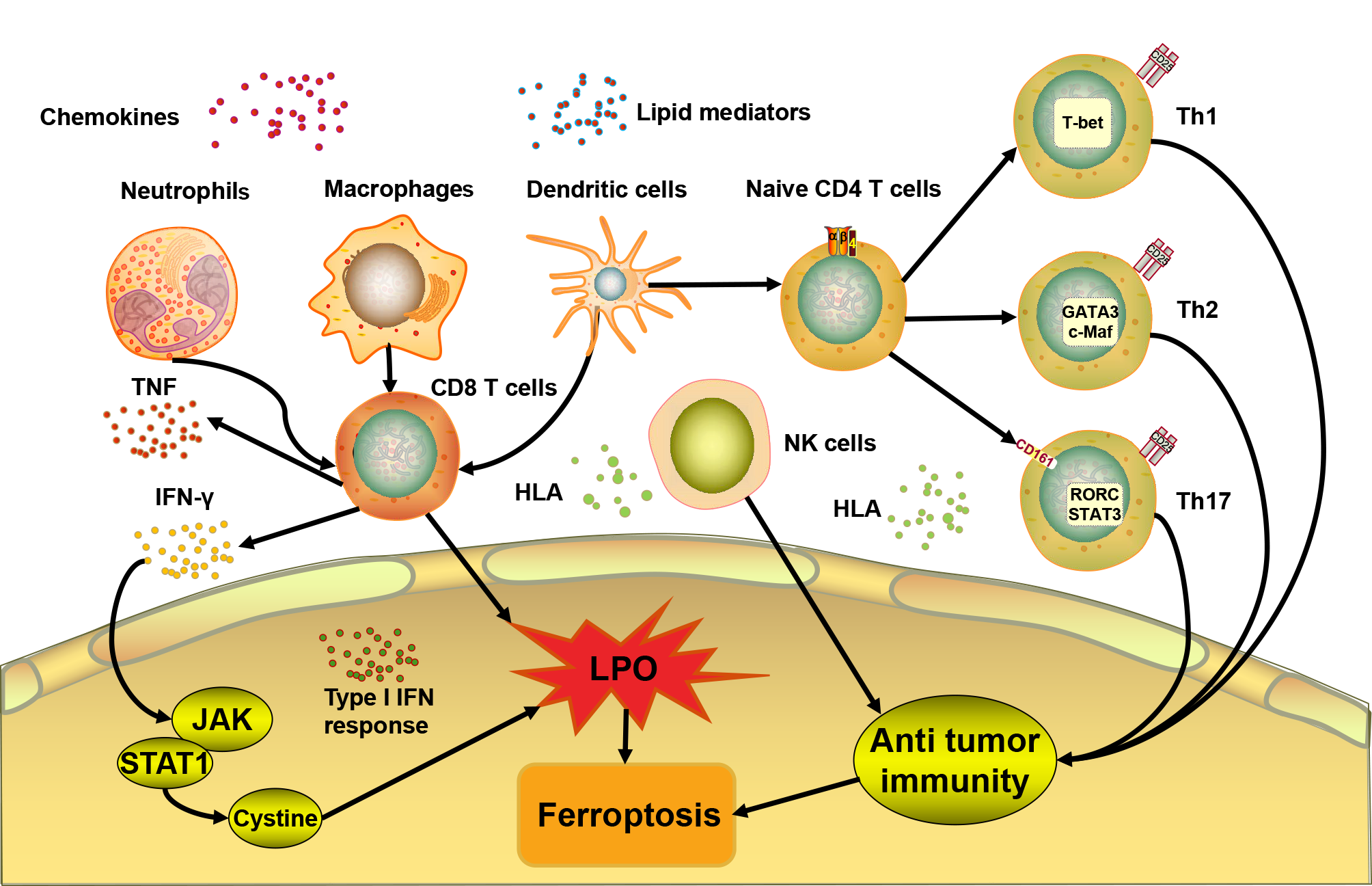
**

**Figure S1.** Crosstalk between ferroptosis and antitumor immunity. Tumor cells undergoing ferroptosis release chemokines and lipid mediators to facilitate the recruitment of APCs, including dendritic cells, macrophages, and neutrophils. CD8+ T cells secret TNF and IFN-γ upon antigen recognition and co-stimulation mediated by APCs. IFN-γactivates JAK/STAT1 signaling pathways and thereby unleashes cystine. This leads to the accumulation of lipid ROS. Dendritic cells mediate co-stimulation of naïve CD4+ T cells to induce T cell activation. Activated T cells differentiate into Th1 cells, Th2 cells, and Th17 cells. These cells together with NK cells further enhance anti-tumor immune response. The level of infiltration of HLA is increased, and type I IFN response is activated during this process. Abbreviation: APC, antigen presenting cell; ROS, reactive oxygen species; Th, T helper; TNF, tumor necrosis factor; IFN, interferon; HLA, human leukocyte antigen; LPO, lipid peroxide.
