# Supplementary material for: A novel ferroptosis-related gene signature for predicting outcomes in cervical cancer
Source: Bioengineered. 2021 May 14;12(1):1813–25. doi: 10.1080/21655979.2021.1925003 (PMC8806749; doi:10.1080/21655979.2021.1925003)
Supplement: Supplemental Material [file KBIE_A_1925003_SM8658.pdf]

The detailed information of 60 ferroptosis-related genes

No.	Gene symbol	Full name
1	ACSL4	Acyl-CoA synthetase long chain family member 4
2	AKR1C1	Aldo-keto reductase family 1 member C1
3	AKR1C2	Aldo-keto reductase family 1 member C2
4	AKR1C3	Aldo-keto reductase family 1 member C3
5	ALOX15	Arachidonate 15-lipoxygenase
6	ALOX5	Arachidonate 5-lipoxygenase
7	ALOX12	Arachidonate 12-lipoxygenase, 12S type
8	ATP5MC3	ATP synthase membrane subunit c locus 3
9	CARS1	Cysteinyl-tRNA synthetase
10	CBS	Cystathionine-beta-synthase
11	CD44	CD44 molecule
12	CHAC1	ChaC glutathione specific gamma-glutamylcyclotransferase 1
13	CISD1	CDGSH iron sulfur domain 1
14	CS	Citrate synthase
15	DPP4	Dipeptidyl peptidase 4
16	FANCD2	Fanconi anemia complementation group D2
17	GCLC	Glutamate-cysteine ligase catalytic subunit
18	GCLM	Glutamate-cysteine ligase modifier subunit
19	GLS2	Glutaminase 2
20	GPX4	Glutathione peroxidase 4

21	GSS	Glutathione synthetase
22	HMGCR	3-hydroxy-3-methylglutaryl-CoA reductase
23	HSPB1	Heat shock protein family B (small) member 1
24	CRYAB	Crystallin alpha B
25	LPCAT3	Lysophosphatidylcholine acyltransferase 3
26	MT1G	Metallothionein 1G
27	NCOA4	Nuclear receptor coactivator 4
28	PTGS2	Prostaglandin-endoperoxide synthase 2
29	RPL8	Ribosomal protein L8
30	SAT1	Spermidine/spermine N1-acetyltransferase 1
31	SLC7A11	Solute carrier family 7 member 11
32	FDFT1	Farnesyl-diphosphate farnesyltransferase 1
33	TFRC	Transferrin receptor
34	TP53	Tumor protein p53
35	EMC2	ER membrane protein complex subunit 2
36	AIFM2	Apoptosis inducing factor, mitochondria associated 2
37	PHKG2	Phosphorylase kinase catalytic subunit gamma 2
38	HSBP1	Heat shock factor binding protein 1
39	ACO1	Aconitase 1
40	FTH1	Ferritin heavy chain 1
41	STEAP3	STEAP3 metalloreductase
42	NFS1	NFS1, cysteine desulfurase

43	ACSL3	Acyl-CoA synthetase long chain family member 3
44	ACACA	Acetyl-CoA carboxylase alpha
45	PEBP1	Phosphatidylethanolamine binding protein 1
46	ZEB1	Zinc finger E-box binding homeobox 1
47	SQLE	Squalene epoxidase
48	FADS2	Fatty acid desaturase 2
49	NFE2L2	Nuclear factor, erythroid 2 like 2
50	KEAP1	Kelch like ECH associated protein 1
51	NQO1	NAD(P)H quinone dehydrogenase 1
52	NOX1	NADPH oxidase 1
53	ABCC1	ATP binding cassette subfamily C member 1
54	SLC1A5	Solute carrier family 1 member 5
55	GOT1	Glutamic-oxaloacetic transaminase 1
56	G6PD	Glucose-6-phosphate dehydrogenase
57	PGD	Phosphogluconate dehydrogenase
58	IREB2	Iron responsive element binding protein 2
59	HMOX1	Heme oxygenase 1
60	ACSF2	Acyl-CoA synthetase family member 2
